# Basal Gnathostomes Provide Unique Insights into the Evolution of Vitamin B12 Binders

**DOI:** 10.1093/gbe/evu289

**Published:** 2014-12-31

**Authors:** Mónica Lopes-Marques, Raquel Ruivo, Inês Delgado, Jonathan M. Wilson, Neelakanteswar Aluru, L. Filipe C. Castro

**Affiliations:** ^1^CIIMAR—Interdisciplinary Centre of Marine and Environmental Research, CIMAR Associate Laboratory, UPorto—University of Porto, Portugal; ^2^ICBAS—Institute of Biomedical Sciences Abel Salazar, UPorto—University of Porto, Portugal; ^3^Department of Biology, Wilfred Laurier University-Waterloo, Ontario, Canada; ^4^Woods Hole Oceanographic Institution, Woods Hole, Massachusetts; ^5^Department of Biology, Faculty of Sciences, UPorto—University of Porto, Portugal

**Keywords:** cobalamin transport, genome duplications, gnathostomes

## Abstract

The uptake and transport of vitamin B12 (cobalamin; Cbl) in mammals involves a refined system with three evolutionarily related transporters: transcobalamin 1 (*Tcn1*), transcobalamin 2 (*Tcn2*), and the gastric intrinsic factor (*Gif*). Teleosts have a single documented binder with intermediate features to the human counterparts. Consequently, it has been proposed that the expansion of Cbl binders occurred after the separation of Actinopterygians. Here, we demonstrate that the diversification of this gene family took place earlier in gnathostome ancestry. Our data indicates the presence of single copy orthologs of the Sarcopterygii/Tetrapoda duplicates *Tcn1* and *Gif,* and *Tcn2*, in Chondrichthyes. In addition, a highly divergent Cbl binder was found in the Elasmobranchii. We unveil a complex scenario forged by genome, tandem duplications and lineage-specific gene loss. Our findings suggest that from an ancestral transporter, exhibiting large spectrum and high affinity binding, highly specific Cbl transporters emerged through gene duplication and mutations at the binding pocket.

## Background

Cobalamin (Cbl; Vitamin B12) is an essential nutrient for metazoans. It is required as the basis for two enzyme cofactors, methyl-Cbl and 5′-deoxyadenosyl-Cbl, for methionine synthase and methyl-malonyl-CoA mutase, respectively, involved in the folate and mutase pathways (Banerjee and Ragsdale 2003). Accordingly, Cbl is fundamental for the synthesis of nucleotides, branched-chain amino acids, and odd-chain fatty acids (Banerjee and Ragsdale 2003; Carmel et al. 2003). Animal diets must include Cbl because only microorganisms are able to synthesize this compound. In humans, Cbl deficiency leads, among others, to anemia and severe neurological dysfunction. Once ingested, an elaborate system involving protein binders is responsible for the absorption, transport, and cellular uptake. Mammalian species typically possess three Cbl binder genes: *Gif* (or gastric intrinsic factor), *Tcn1* (also known as haptocorrin), and *Tcn2* (also known as transcobalamin). This carrier diversity mirrors their specialization to different physiological environments and functional specificities. Interestingly, *Tcn1* is absent in some species such as mouse, rat, and probably the marsupial opossum (Greibe, Fedosov, Nexø, et al. 2012). In birds and amphibians two binders have been found, whereas in reptiles a similar gene repertoire to that of most mammals is found (Greibe, Fedosov, Nexø, et al. 2012). Teleosts such as zebrafish, trout, and salmon have a single documented binder (Greibe, Fedosov, Nexø, et al. 2012; Greibe, Fedosov, Sorensen, et al. 2012). This has led to the proposal that the evolutionary elaboration of Cbl binding proteins occurred “after” the divergence of Actinopterygians (Greibe, Fedosov, Nexø, et al. 2012). In effect, the zebrafish and trout Cbl binding protein exhibits mixed characteristics. Structurally it resembles a hybrid of the full set of human Cbl binders. The sequence identity is closer to *Tcn2* (Greibe, Fedosov, Nexø, et al. 2012), the amino acid composition at the binding site is similar to *Tcn1* (Greibe, Fedosov, Nexø, et al. 2012; Greibe, Fedosov, Sorensen, et al. 2012), and it shows resistance toward degradation by trypsin comparable to *Gif* (Greibe, Fedosov, Nexø, et al. 2012; Greibe, Fedosov, Sorensen, et al. 2012). Consequently, it has been named *HIT* (an abbreviation for haptocorrin, intrinsic factor, and transcobalamin) denoting its intermediate and ancestral nature (Greibe, Fedosov, Nexø, et al. 2012).

To infer the exact evolutionary history of a gene family based on functional aspects, without considering phylogenetics and an adequate species sampling, can lead to inaccurate conclusions. Particularly relevant when addressing these issues is the role of gene/genome duplications and gene loss. For example, two rounds of whole-genome duplications (1R and 2R) have taken place in early vertebrate ancestry (Putnam et al. 2008). Additional events of whole-genome duplication have occurred, one in teleost ancestry (3R) (Jaillon et al. 2004), and a second specifically in salmonids (4R) (Berthelot et al. 2014). In this context, the repertoire of Cbl binding proteins in teleosts may represent a case of secondary lineage specific-gene loss after duplication and not an ancestral state.

Here, to distinguish between different evolutionary hypotheses, we analyzed the gene diversity and *loci* composition in a variety of vertebrate species, particularly in basal gnathostomes, Chondrichthyans.

## Materials and Methods

### Sequence Mining and Phylogenetic Analysis

*Tcn1*, *Gif*, and *Tcn2* sequences from all major vertebrate lineages and from the invertebrate species *Branchiostoma floridae* (amphioxus) and *Saccoglossus kowalevskii* (acorn worm) were identified in the Ensembl, GenBank, Skatebase (http://skatebase.org/) databases via tBLASTn and BLASTp searches using as reference annotated human Cbl binder sequences. Amino acid sequences were aligned with MAFFT alignment software (Katoh and Toh 2010) using default parameters and visualized and edited in Geneious v7.1.7. *Gallus gallus* TCN2 partial sequence (XP_427292.3) was excluded from the analysis. Although extensive searches were performed, we were unable to retrieve *Chelonia mydas Gif* and *Tcn1, Pelodiscus sinensis Tcn2* and *Gif*, as well as *Taeneopygia guttata Tcn2* possibly due to poor genome coverage. To infer the evolutionary model (LG + G) used for phylogenetic analysis, the alignment was stripped from columns containing gaps resulting in an alignment with 268 positions which was analyzed in Protest 3.3 (Abascal et al. 2009). Finally, phylogenetic analysis was performed on the online platform PhyML 3.0 (http://www.atgc-montpellier.fr/phyml/), and the aBayes algorithm was selected to calculate branch support (Guindon et al. 2010).

### Comparative Genomics

*Tcn1, Gif,* and *Tcn2* genes were localized onto the human chromosomes, the location of each gene and the neighboring genes were collected from Ensembl and GenBank databases. Gene *loci* in human were used as a reference to assemble the synteny maps of the remaining species. Gene families with multiple members (e.g., oxysterol binding protein - OSBP) flanking these genes in humans had their phylogenetic history determined to clarify if the duplication timing coincided with 2R (not shown).

### Gene Isolation and Expression Analysis

Adult *Leucoraja erinacea* were obtained from the Marine Biological Laboratory’s Marine Resources Center in Woods Hole, Massachusetts. Fish were collected from the coast of Woods Hole and maintained in 100 gallon recirculatory tanks under ambient conditions. All tissues were collected and preserved in RNAlater and stored at −20 °C. Total RNA was isolated using an Illustra RNAspin Mini RNA Isolation Kit (GE Healthcare, UK) according to the manufacturer's recommendations, including the on-column treatment of isolated RNA with RNase-free DNase I. RNA concentration was calculated using Qubit fluorometer instrument (Invitrogen, Carlsbad CA), integrity confirmed by electrophoresis and the RNA stored at −80 °C until further use. Partial Tcn-like sequences were extended by Rapid amplification of cDNA ends (RACE) polymerase chain reaction (PCR) with the SMARTer 5′/3′ Kit (Clontech). *Leucoraja erinacea* full (or near full) open reading frames (ORFs) were obtained by PCR with the following primer sets: *Tcn1/Gif* primer forward 5′-GGGCAAGCAGTGGTATCAAC-3′, primer reverse 5′-GTTAGAGCGATGGGGAGAGG-3′; *Tcn3* primer forward 5′-ACGCAGAGTACATGGGGACT-3′, primer reverse 5′-TTATTAGTTGGCGGCGTTTC-3′ and *Tcn2* primer forward 5′-AGTGTCCACATTGCCTTGC-3′, primer reverse 5′-CCTGTAATTTGGGGCTTTCA-3. The cDNA was synthesized from 500 ng of total RNA with the iScript cDNA Synthesis Kit (Bio-Rad) according to the manufacturer's protocol. Tissue expression was determined through RT-PCR with intron flanking primers. PCR was performed using 2 μl of little skate cDNA and Phusion Flash high-fidelity Master Mix (FINNZYMES). PCR parameters were as follows: initial denaturation at 98 °C for 10 s, followed by 35 cycles of denaturation at 98 °C for 1 s, annealing for 5 s and elongation at 72 °C for 10 s, and a final step of elongation at 72 °C for 1 min. PCR products were then loaded onto 2% agarose gel stained with GelRed and run in TBE buffer at 80 V.

### Comparative Homology Modeling

*Tcn1/Gif*, *Tcn2*, and *Tcn3* amino acid sequences of *L. erinacea* were submitted to the online platform iTASSER (Zhang 2008; Roy et al. 2010) for modeling. The predicted structural models were analyzed and visualized using Open-Source PyMOL V1.3 (academic version; Schrödinger 2011).

## Results

**Fig. 1.—:**
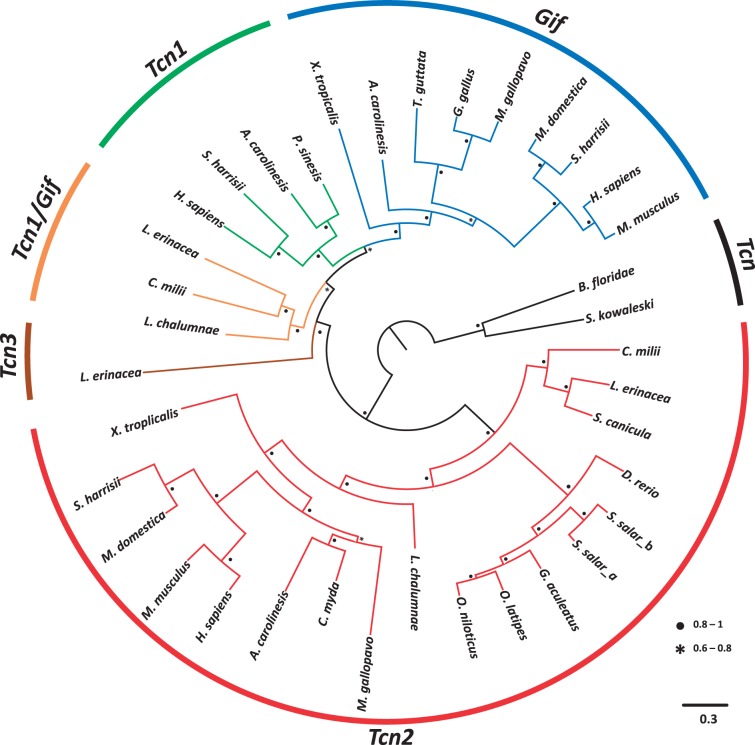
Maximum likelihood phylogenetic tree describing relationships among Cbl binding proteins from representative vertebrate *taxa* and two invertebrate deuterostomes. Node values represent branch support using the aBayes algorithm. Accession numbers for all sequences are provided in the supplementary material, Supplementary Material online.

**Fig. 2.—:**
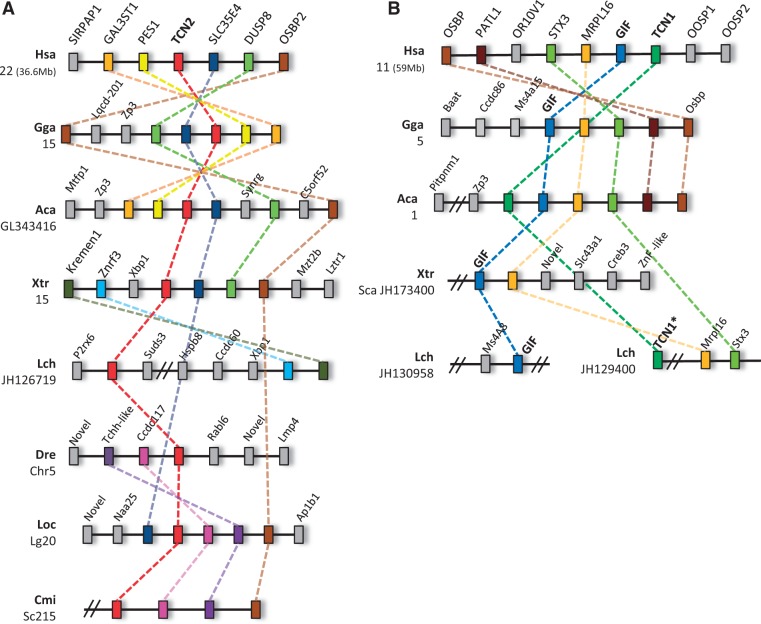
Synteny maps of *Tcn1*, *Gif*, and *Tcn2 loci*. (*A*) Detail of the *Tcn2 locus* which is highly conserved in major vertebrate lineages; (*B*) Detail of the *Tcn1* and *Gif locus*, depicting a highly conserved *locus* in tetrapods. The *Tcn1 locus* is disrupted in teleosts (not shown). Information is presently absent for the *Tcn1* and *Tcn3 loci* in the chondrichthyan lineage. Hsa, *Homo sapiens*; Gga, *Gallus gallus*; Aca, *Anolis carolinensis*; Xtr, *Xenopus tropicalis*; Lch, *Latimeria chalumnae*; Dre, *Danio rerio*; Loc, *Lepisosteus oculatus*; and Cmi, *Callorhinchus milii*. * denotes partial sequence. Double dashes denote gap.

We began by providing clarification of the orthology of the teleost single copy sequences. Our analysis shows that the teleost Cbl binder forms a monophyletic clade with Sarcopterygii *Tcn2* (fig. 1). Further evidence for the common origin of *Tcn2* sequences comes from synteny analysis (fig. 2). The *Tcn2 locus* is relatively well conserved in the examined species, with the exception of zebrafish (fig. 2). However, the holostean spotted gar *Tcn2 locus* (*Lepisosteus oculatus*), which diverged prior to the teleost-specific duplication (3R), retains synteny with tetrapods (e.g., *SLC35E4*; fig. 2). Thus, teleost sequences should be named *Tcn2* and not *HIT*. Additionally, we also find a novel *Tcn2* gene specific to salmon, probably resulting from the salmonid-specific genome duplication, 4R (fig. 1).

The branching pattern of our phylogenetic analysis provides additional clues on the probable timing of *Tcn1* and *Gif* emergence. Orthologs of both genes can be found in mammals (except *Tcn1* in rodents and the opossum) and reptiles. In contrast, amphibians and birds have a single gene but clearly grouping with other *Gif* sequences (fig. 1). In the basal Sarcopterygian coelacanth we found two *Gif/Tcn1-like* sequences. However, one is an incomplete sequence too short to include in the phylogenetic analysis (not shown; supplementary material, Supplementary Material online). The basal position of the complete coelacanth sequence in the tree suggests that *Gif* and *Tcn1* originated from a duplication event in the ancestor of Tetrapoda (fig. 1). However, the incomplete Cbl binder sequence of the coelacanth is flanked by genes whose orthologs in tetrapods localize to the *Gif* and *Tcn1* genomic location (fig. 2). Thus, both sequences might represent bona fide *Gif* and *Tcn1* orthologs. The completion of the partial coelacanth sequence as well as the investigation of the Cbl binder gene repertoire in lungfish should help to resolve this matter. Additionally, the syntenic composition of this *locus* in various lineages (fig. 2) in combination with the phylogenetic analysis supports the independent loss of *Tcn1* in amphibians, birds, and some mammalian species.

The overall evolutionary branching pattern suggests that the *Tcn2* and *Tcn1*/*Gif* gene duplication predates teleost radiation (fig.1), and so a *Gif/Tcn1* gene would have been independently lost in this lineage. Interestingly, additional *Tcn*-like sequences have been reported in teleosts (Greibe, Fedosov, Nexø, et al. 2012). These are shorter than typical TCN proteins, composed of a DUF4430 domain present in the C-terminus region, similarly to TCN proteins (supplementary material, Supplementary Material online). Although they could represent the remnants of an ancestral *Gif/Tcn1* gene(s), neither phylogenetics (not shown) nor synteny analysis (supplementary material, Supplementary Material online) clarifies their origin.

To further explore the evolutionary history of Cbl binders, we next investigated the gene repertoire in the most basal clade of jawed vertebrates, the Chondrichthyans. In the recent release of the elephant shark genome sequence, we identified an ortholog of *Tcn2* (fig. 1; Venkatesh et al. 2014). Furthermore, a second sequence was found in the transcriptome of the same species which branches basally to the *Tcn1* and *Gif* clade (fig.1), thus supporting an event of gene loss in the Actinopterygii lineage. We next examined the partial genome and transcriptome sequences of the little skate, *L. erinacea* (Wang et al. 2012). Surprisingly, we found three partial sequences with similarity to Cbl binders, which were further expanded by PCR to obtain full or near full-length sequences. Phylogenetic analysis indicates that two of these group with the *Tcn2* and *Tcn1*/*Gif* gene clades, respectively, as observed in the elephant shark (fig. 1), while the third represents an apparently novel Cbl gene lineage so far unique to little skate, which we name *Tcn3* (fig. 1). To envisage the evolutionary origin of this extra sequence without synteny data, which is currently unavailable for this species, is problematic. Nevertheless, paralogy analysis of the human *loci* containing *Tcn2* and *Tcn1/Gif* provides a plausible explanation (supplementary material, Supplementary Material online). In effect, these genes reside in genomic regions related by duplication dating back to 2R (Putnam et al. 2008). For example, the *OSBP* gene, which maps close to human *Tcn1*/*Gif* at chromosome 11 has a paralog, *OSBP2*, close to *Tcn2* at chromosome 22 (supplementary material, Supplementary Material online). Detailed analysis shows various gene families whose paralogs map in expected regions of paralogy (supplementary material, Supplementary Material online), thus indicating that *Tcn2* and the ancestor of *Tcn1* and *Gif* are 2R-generated paralogs. In this context, we put forward that *Tcn3* might represent a 2R paralog retained uniquely in Elasmobranchii (or Chondrichthyans) but subsequently lost in other gnathostome lineages, similarly to what has been described in other gene families (e.g., Mulley and Holland 2010; Hoffmann et al. 2011, 2012; Ravi et al. 2013). In an alternative scenario the Elasmobranchii *Tcn3* and *Tcn1*/*Gif* genes might represent true *Gif* and *Tcn1* orthologs respectively, whose phylogenetic relationships toward Sarcopterygii sequences have been obscured by sequence divergence. If so, the duplication of *Tcn1* and *Gif* would date back to the origin of gnathostomes. Interestingly, the expression of the so-called *Tcn3* in little skate is significantly higher in the stomach (supplementary material, Supplementary Material online), paralleling the mammalian *Gif*. Whether *Tcn3* is present in other Chondrichthyes species is also a pertinent question, in particular the elephant shark given its agastric condition (Castro et al. 2014). Further investigations, namely with the inclusion of synteny data should fully clarify the origin of *Tcn3*.

## Discussion

**Fig. 3.—:**
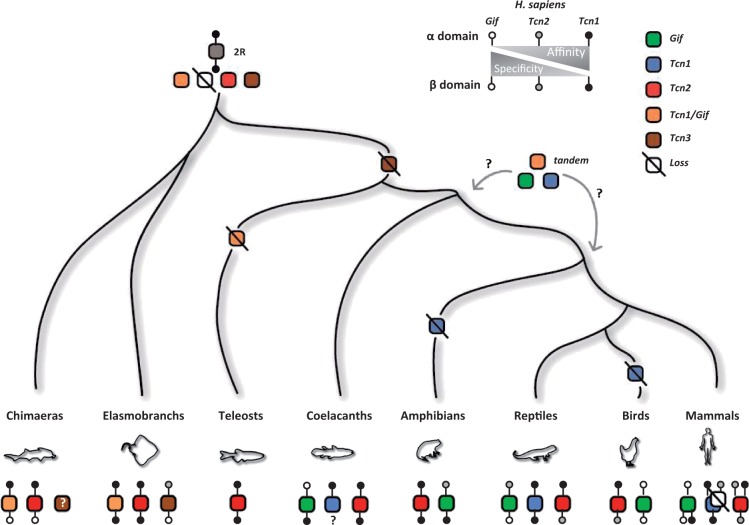
Evolutionary model of Cbl binding proteins in vertebrates. Specificity and affinity gradients illustrate the binding properties of the human carriers (top). Grayscale circles indicate α and β domain signature motif conservation in vertebrate carriers deduced from table 1 (bottom).

In this study, we explored the evolutionary history of Cbl binding proteins in vertebrates. Our findings support a model where a single Cbl binder duplicated in early vertebrate ancestry as part of 2R (fig. 3). A later event of duplication (tandem) in the ancestor of either Sarcopterygii or Tetrapoda gave origin to *Gif* and *Tcn1*, with the latter being lost independently in amphibians, birds, and some mammalian species (fig. 3). In teleosts only *Tcn2* has been retained. We also found a novel, highly divergent Cbl binder in little skate, *Tcn3*, even though without synteny data we cannot firmly conclude on its evolutionary origin. Why exactly have different lineages retained such a variable repertoire of Cbl binders is difficult to establish *a priori*. Although all binders share a similar structure, they display distinct physiological functions. In mammals, the specificity toward Cbl is higher for GIF and TCN2 but substantially lower for TCN1 (Fedosov et al. 2007). In fact, gastric GIF and plasma TCN2 are required for Cbl absorption via receptor-mediated endocytosis in the ileum and target cells, respectively (Furger et al. 2012). TCN1, on the other hand, occurs in several body fluids, including saliva, milk, and plasma (Morkbak et al. 2007). When compared with the other carriers, TCN1 exhibits higher binding affinity toward Cbl, faster binding kinetics, and lower specificity, binding other apparently inert corrinoids (Fedosov et al. 2007; Wuerges et al. 2007). In the lumen of the upper gut, TCN1 confers increased stability to the carrier-corrinoid complex at low pH conditions; yet, due to its reduced resistance to pancreatic proteolytic enzymes Cbl is relayed to GIF in the small intestine (Greibe, Fedosov, Sorensen, et al. 2012). Although TCN1 also binds the vast majority of Cbl in the plasma, its role there is more elusive. Given its glycosylation status (Wuerges et al. 2007; Furger et al. 2012), TCN1-dependent corrinoid uptake from plasma via liver asialoglycoprotein receptors was suggested (Furger et al. 2012). Thus, TCN1 likely recycles Cbl, to an additional round of intestinal absorption, as well as acts as a scavenger in the blood for toxic Cbl-derived molecules, leading to its excretion (Wuerges et al. 2007). Interestingly, this later capacity is not unique to TCN1. The lack of TCN1 in mice is apparently functionally compensated by the action of TCN2 (Hygum et al. 2011). For example, the murine TCN2 is capable of binding to a Cbl analogue, cobinamide, just like the human TCN1 transporter (Hygum et al. 2011). Similarly, in zebrafish, TCN2 has been found to bind the analogue cobinamide, while still efficiently binding Cbl (Greibe, Fedosov, Nexø, et al. 2012). This functional plasticity is apparently structurally determined (table 1). Despite poor sequence conservation Cbl carriers retain a similar two domain-structure, α and β, that clamp corrinoids (Wuerges et al. 2007), also visible in the three little skate carriers (supplementary material, Supplementary Material online). In humans, several features apparently sustain their differential affinity and selectivity: interdomain contacts and complementarity and carrier-specific ligand interactions (Wuerges et al. 2007; Furger et al. 2013) (table 1; supplementary material, Supplementary Material online). However, some reported structural differences are human specific and cannot be transposed to other groups (e.g. an additional disulfide bridge in human TCN1). The lower specificity and higher affinity of human TCN1 were justified by the presence of two amino acid clusters (table 1; supplementary material, Supplementary Material online; Wuerges et al. 2007; Furger et al. 2013). On the β-domain three bulky residues (Arg^357^, Trp^359^, and Tyr^362^), the first human specific, provide hydrophobic contacts and stabilize the TCN1-corrinoid complex, compensating for the lack of the nucleotide moiety in cobinamide, a baseless corrinoid (Wuerges et al. 2007). Human GIF and TCN2 exhibit single bulky residues at distinct positions, Trp^359^ or Tyr^362^, respectively. The second motif, on the α-domain (TNYYQ), was suggested to form four H-bonds with the central corrinoid ring; a decreasing number of possible H-bonds is observed in TCN2 and GIF, corroborating the decline in the thermal stability of the carrier-corrinoid complexes and the gradual decrease of affinity toward Cbl (TCN1>TCN2>GIF) (Furger et al. 2013). Thus, TCN1 has been suggested to act as a scavenger in the blood for toxic Cbl-derived molecules (Wuerges et al. 2007). The occurrence of these motifs seems highly plastic throughout gnathostome evolution; yet scavenger-like carriers, retaining one or both amino acid motif signatures, are observed in all the examined species, including in the preduplicated TCN of cephalochordates (table 1; supplementary material, Supplementary Material online). For instance, in mouse, TCN2 retains the TNYYQ binding motif, but not the pair of bulky residues (table 1). Nonetheless, this carrier functionally behaves like TCN1: exhibiting an affinity toward Cbl comparable to that of human TCN1 and ability to bind cobinamide, yet with lower efficiency (Hygum et al. 2011). Similar results were obtained with teleost TCN2 (Greibe, Fedosov, Nexø, et al. 2012), which appears to display the full set of motifs, with the Tyr residue of the β-domain replaced by a similarly bulky Phe (table 1). Marsupials and birds, on the other hand, lack a true TNYYQ-like carrier but retain the second motif of aromatic residues (table 1). Thus, it is plausible to hypothesize that these carriers are able to bind and stabilize corrinoids, other than Cbl, to some extent. Although ligand recognition alone does not fully account for the variable number of Cbl carriers in the examined species, it illustrates the plasticity of these proteins (fig. 3). Overall, we suggest that from an ancestral protein with high affinity but low specificity toward Cbl, the increase in binding specificity was acquired (and lost) through gene duplication and recurrent mutations at the carrier Cbl binding pocket (fig. 3 and table 1). Conversely, carriers exhibiting large spectrum and high affinity binding seem persistent, as are some binders with mixed profiles (fig. 3). The ancestral condition, retained in teleost and Elasmobranchii TCN2, shifted to additional carriers upon duplication events, as seen in most mammalian and reptile TCN1. In agreement, mouse TCN2 recapitulates the ancestral phenotype upon TCN1 loss. This functional shift possibly paralleled the acquisition of novel features in mammalian Cbl metabolism, notably membrane receptor recognition in target cells (Quadros et al. 2009). Our findings illustrate the decisive importance of basal gnathostomes to clarify gene family evolution and physiological diversity.

**Table 1:** Cross-Species Variation of α and β Cbl Domain Signature Motifs

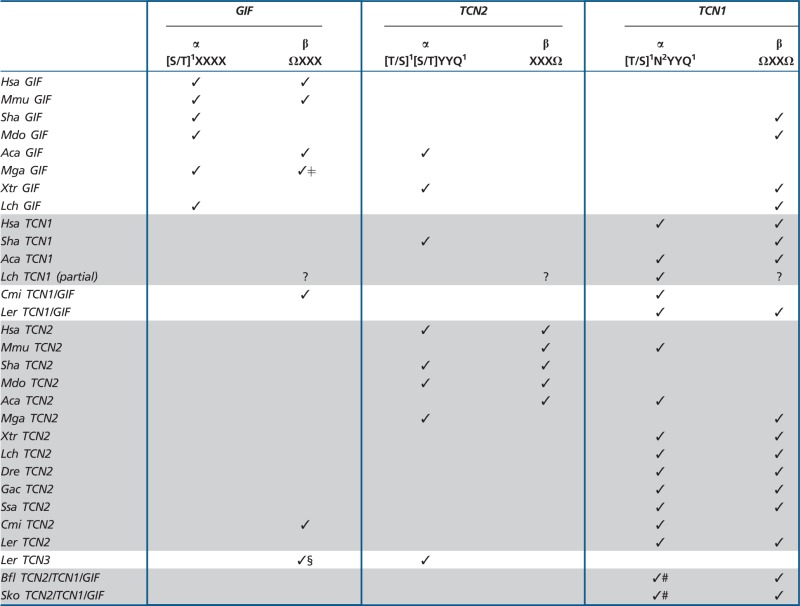

Note.—The number of H-bonds formed between the α–domain of the human carriers and the corrinoid ring are indicated in superscript (^1/2^). Ω represents the bulky hydrophobic residues of the β-domain. Gradual shifts in human carrier affinity and specificity are represented in the diagram above. §, ΩXΩX; ǂ, XXXX; #, NNXXQ; ?, unkown. Hsa, *Homo sapiens*; Mmu, *Mus musculus*; Sha, *Sarcophilus harrisii*; Mdo, *Monodelphis domestica*; Aca, *Anolis carolinensis*; Mga, *Meleagris gallopavo*; Xtr, *Xenopus tropicalis*; Lch, *Latimeria chalumnae*; Cmi, *Callorhinchus milii*; Ler, *Leucoraja erinacea*; Dre, *Danio rerio*; Gac, *Gasterosteus aculeatus*; Ssa, *Salmo salar*; Bfl, *Branchiostoma floridae*; and Sko, *Saccoglossus kowalevskii*.

## Supplementary Material

Supplementary material is available at *Genome Biology and Evolution* online (http://www.gbe.oxfordjournals.org/).

Supplementary Data
