# Supplementary material for: Basal Gnathostomes Provide Unique Insights into the Evolution of Vitamin B12 Binders
Source: Genome Biol Evol. 2014 Dec 31;7(2):457–64. doi: 10.1093/gbe/evu289 (PMC4350170; doi:10.1093/gbe/evu289)
Supplement: Supplementary Data [file supp_7_2_457__index.html]

Basal gnathostomes provide unique insights into the evolution of vitamin B12 binders — Basal Gnathostomes Provide Unique Insights into the Evolution of Vitamin B12 Binders — Supplementary Data 

# Basal Gnathostomes Provide Unique Insights into the Evolution of Vitamin B12 Binders

## Supplementary Data

files

**Files in this Data Supplement:**

- Supplementary Data - docx file
