# Supplementary material for: Basal Gnathostomes Provide Unique Insights into the Evolution of Vitamin B12 Binders
Source: Genome Biol Evol. 2014 Dec 31;7(2):457–64. doi: 10.1093/gbe/evu289 (PMC4350170; doi:10.1093/gbe/evu289)
Supplement: Supplementary Data [file supp_evu289_Supplementary_materialGBE.docx]

**Supplementary Material**

**Supplementary table 1**. List of sequences used for phylogenetic analysis.

| Species | **Gene** | **Accession number** |  | **Species** | **Gene** | **Accession number** |
| --- | --- | --- | --- | --- | --- | --- |
| *Anolis carolinensis* | Tcn1 | XP_003215131.1 |  | ***Monodelphis domestica*** | Tcn2 | XP_001380461.1 |
|  | Tcn2 | XP_008117996.1 |  |  | Gif | XP_007497607.1 |
|  | Gif | XP_008108890.1 |  | ***Meleagris gallopavo*** | Tcn2 | ENSMGAP00000015262 |
| *Branchiostoma floridae* | Tcn | XP_002605724.1 |  |  | Gif | ENSMGAP00000003005 |
| *Callorhinchus milii* | Tcn1/Gif | JW870881.1 |  | ***Mus musculus*** | Tcn2 | NP_056564.1 |
|  | Tcn2 | NP_001279906.1 |  |  | Gif | NP_032144.2 |
| *Chelonia mydas* | Tcn2 | EMP27579.1 |  | ***Oryzias latipes*** | Tcn2 | XP_004072417.1 |
| *Danio rerio* | Tcn2 | NP_001116703.1 |  | ***Oreochromis niloticus*** | Tcn2 | XP_005473430.1 |
| *Gasterosteus aculeatus* | Tcn2 | ENSGACP00000011296 |  | ***Pelodiscus sinesis*** | Tcn1 | XP_006110368.1 |
| *Gallus gallus* | Gif | XP_001233885.2 |  | ***Scyliorhinus canicula*** | Tcn2 | SSC-transcript-ctg15613 |
| *Homo sapiens* | Tcn1 | NP_001053.2 |  | ***Sarcophilus harrisii*** | Tcn1 | XP_003774045.1 |
|  | Tcn2 | NP_000346.2 |  |  | Tcn2 | XP_003762764.1 |
|  | Gif | NP_005133.2 |  |  | Gif | XP_003774041.1 |
| *Latimeria chalumnae* | Gif | XP_006013605.1 |  | ***Salmo salar*** | Tcn2 a | NP_001133733.1 |
|  | Tcn1  partial | XP_006011707.1 |  |  | Tcn2 b | ACN10392.1 |
|  | Tcn2 | XP_005993347.1 |  | ***Saccoglossus kowaleski*** | Tcn | XP_002734140.1 |
| *Leucoraja erinacea* | Tcn1/ Gif | KP273228 |  | ***Taeniopygia guttata*** | Gif | XP_002196005.2 |
|  | Tcn2 | KP273226 |  | ***Xenopus tropicalis*** | Tcn2 | NP_001184035.1 |
|  | Tcn3 | KP273227 |  |  | Gif | XP_002941420.1 |

**Supplementary figures**

**Hs_TCN1_NP_001053.2**  1 MRQS------ ---------- HQLPLVGLLL FSF--I-PSQ LCEICEVSEE NYIRLKPL-- ---------- ---LNTMIQS 46

**Hs_GIF_NP_005133.2**  1 -MAW------ ---------- FALYLLSLLW ATA--GTSTQ TQSSCSVPSA QEPLVNGI-- ---------- ---QVLMENS 46

**Hs_TCN2_NP_000346.2**  1 ---------- ---------- --MRHLGAFL FLL--GVLGA LTEMCEIPEM DSHLVEKLGQ HLLPWMDRLS LEHLNPSIYV 56

**Medaka_ENSORLT00000019254**  1 ---------- ---------- --MKEPALIA AAL--L---- ---------- ---------- ---------- ---LLLPAAL 19

**Medaka_ENSORLT00000001408**  1 MAFR------ ---------- VIFTAAVLIL LTQ--AGDEG DDEGANSDRG SHAGAAAL-- ---------- ---LRHSDTV 47

**Tilapia_ENSONIT00000013242**  1 ---------- ---------- ---------- ---------- ---------- ---------- ---------- ---------- 1

**Tetradon_ENSTNIT00000017613**  1 ---------- ---------- ---MKLVLLS AAL--L---- ---------- ---LLLPA-- ---------- ---ARPERHQ 23

**Cavefish_ENSAMXT00000002854**  1 ---------- ---------- ---------- ---------- ---------- ---------- ---------- ---MPPTAFT 7

**Stickleback_ENSGACT00000027033** 1 ---------- ---------- MAPRTAALLS AGF--L---- ---------- ---LL----- ---------- ---LTQRALT 23

**Platyfish_ENSXMAT00000005953**  1 MRAWSSINTD VKPAGETSHR MAPRLAAQLS VSFLLL---- ---------- ---LL----- ---------- ---LPHEAIT 45

**Amazonmolly_ENSPFOT00000010148** 1 MRGWSSINTD VKPGGETSHR MAPRPAALLS VSF-LL---- ---------- ---LL----- ---------- ---LPHEAIA 44

**Tilapia_ENSONIT00000006763**  1 ---------- ---------- MALRTSALLS VGF--F---- ---------- ---LL----- ---------- ---LTCGALT 23

**Tetraodon_ENSTNIT00000014896**  1 ---------- ---------- MRLRT-PLLS VGV--L---- ---------- ---LL----- ---------- ---LTGGVLT 22

**Codfish_ENSGMOT00000005936**  1 ---------- ---------- ---------- ---------- ---------- ---------- ---------- ---------- 1

**Cavefish_ENSAMXT00000003614**  1 ---------- ---------- LALSLVIFLC STV--L---- ---------- ---ICEPA-- ---------- ---LHIRAIQ 26

**Zebrafish_ENSDART00000098273**  1 ---------- ---------- MALTAISLLC FTA--L---- ---------- ---LCFPA-- ---------- ---LGLPADS 26

**Zebrafish_ENSDART00000147769**  1 ---------- ---------- MTLTAITLLC FAA--L---- ---------- ---LPFPG-- ---------- ---LGKGGHS 26

**Spottedgar_ENSLOCT00000004606**  1 ---------- ---------- MAL-AVTMIL STV--L---- ---------- ---LLVPA-- ---------- ---LLVQPES 25

**Spottedgar_ENSLOCT00000004639**  1 ---------- ---------- MAL-AVTMIL STV--L---- ---------- ---LLVPA-- ---------- ---LLVQAES 25

**Tilapia_ENSONIT00000020351**  1 ---------- ---------- --MKKPALLS AVL------- ---------- ---LLLFF-- ---------- ---VGTSAQG 23

**Hs_TCN1_NP_001053.2**  47 NYNRGTSAVN VVLSLKLVGI QIQTLMQKMI QQIKYNVKSR LSDVSSGELA LIILALGVCR NAEENLIYDY HLIDKLENKF 126

**Hs_GIF_NP_005133.2**  47 VTSSAYPNPS ILIAMNLAGA YNLKAQKLLT YQLMSSDNND ---LTIGQLG LTIMALTSSC ---------R DPGDKVSILQ 114

**Hs_TCN2_NP_000346.2**  57 GLRLSSLQAG TKEDLYLHSL KLGYQQCLLG SAFSEDDGDC QGKPSMGQLA LYLLALRANC EFVRGHKGDR LVSQLKWFLE 136

**Medaka_ENSORLT00000019254**  20 TQNF------ ---------- ---------- ---------- ---------- ---------- ---------- ---------- 23

**Medaka_ENSORLT00000001408**  48 RQDN------ ---------- ---------- ---------- ---------- ---------- ---------- ---------- 51

**Tilapia_ENSONIT00000013242**  1 ---------- ---------- ---------- ---------- ---------- ---------- ---------- ---------- 1

**Tetradon_ENSTNIT00000017613**  24 RGSV------ ---------- ---------- ---------- ---------- ---------- ---------- ---------- 27

**Cavefish_ENSAMXT00000002854**  8 SDVE------ ---------- ---------- ---------- ---------- ---------- ---------- ---------- 11

**Stickleback_ENSGACT00000027033** 24 ETGP------ ---------- ---------- ---------- ---------- ---------- ---------- ---------- 27

**Platyfish_ENSXMAT00000005953**  46 NQGL------ ---------- ---------- ---------- ---------- ---------- ---------- ---------- 49

**Amazonmolly_ENSPFOT00000010148** 45 NQGL------ ---------- ---------- ---------- ---------- ---------- ---------- ---------- 48

**Tilapia_ENSONIT00000006763**  24 DTDE------ ---------- ---------- ---------- ---------- ---------- ---------- ---------- 27

**Tetraodon_ENSTNIT00000014896**  23 NAGP------ ---------- ---------- ---------- ---------- ---------- ---------- ---------- 26

**Codfish_ENSGMOT00000005936**  1 ---------- ---------- ---------- ---------- ---------- ---------- ---------- ---------- 1

**Cavefish_ENSAMXT00000003614**  27 TEEL------ ---------- ---------- ---------- ---------- ---------- ---------- ---------- 30

**Zebrafish_ENSDART00000098273**  27 GKLE------ ---------- ---------- ---------- ---------- ---------- ---------- ---------- 30

**Zebrafish_ENSDART00000147769**  27 GEQH------ ---------- ---------- ---------- ---------- ---------- ---------- ---------- 30

**Spottedgar_ENSLOCT00000004606**  26 S--------- ---------- ---------- ---------- ---------- ---------- ---------- ---------- 26

**Spottedgar_ENSLOCT00000004639**  26 SEAR------ ---------- ---------- ---------- ---------- ---------- ---------- ---------- 29

**Tilapia_ENSONIT00000020351**  24 NTNF------ ---------- ---------- ---------- ---------- ---------- ---------- ---------- 27

**Hs_TCN1_NP_001053.2**  127 QAEIENMEAH NGTPLTNYYQ LSLDVLALCL FNGNYSTAEV VNHFTPENKN YYFGSQFSVD TGAMAVLALT CVKKSLINGQ 206

**Hs_GIF_NP_005133.2**  115 RQMENWAPSS PNAEASAFYG PSLAILALCQ KNSEATLPIA VRFAKTLLAN ---SSPFNVD TGAMATLALT CMYNKI---P 188

**Hs_TCN2_NP_000346.2**  137 DEKRAIGHDH KGHPHTSYYQ YGLGILALCL HQKRVHDSVV DKLLYAVEPF H--QGHHSVD TAAMAGLAFT CLKRS----- 209

**Medaka_ENSORLT00000019254**  23 ---------- ---------- ---------- ---------- ---------- ---------- ---------- ---------- 23

**Medaka_ENSORLT00000001408**  51 ---------- ---------- ---------- ---------- ---------- ---------- ---------- ---------- 51

**Tilapia_ENSONIT00000013242**  1 ---------- ---------- ---------- ---------- ---------- ---------- ---------- ---------- 1

**Tetradon_ENSTNIT00000017613**  27 ---------- ---------- ---------- ---------- ---------- ---------- ---------- ---------- 27

**Cavefish_ENSAMXT00000002854**  11 ---------- ---------- ---------- ---------- ---------- ---------- ---------- ---------- 11

**Stickleback_ENSGACT00000027033** 27 ---------- ---------- ---------- ---------- ---------- ---------- ---------- ---------- 27

**Platyfish_ENSXMAT00000005953**  49 ---------- ---------- ---------- ---------- ---------- ---------- ---------- ---------- 49

**Amazonmolly_ENSPFOT00000010148** 48 ---------- ---------- ---------- ---------- ---------- ---------- ---------- ---------- 48

**Tilapia_ENSONIT00000006763**  27 ---------- ---------- ---------- ---------- ---------- ---------- ---------- ---------- 27

**Tetraodon_ENSTNIT00000014896**  26 ---------- ---------- ---------- ---------- ---------- ---------- ---------- ---------- 26

**Codfish_ENSGMOT00000005936**  1 ---------- ---------- ---------- ---------- ---------- ---------- ---------- ---------- 1

**Cavefish_ENSAMXT00000003614**  30 ---------- ---------- ---------- ---------- ---------- ---------- ---------- ---------- 30

**Zebrafish_ENSDART00000098273**  30 ---------- ---------- ---------- ---------- ---------- ---------- ---------- ---------- 30

**Zebrafish_ENSDART00000147769**  30 ---------- ---------- ---------- ---------- ---------- ---------- ---------- ---------- 30

**Spottedgar_ENSLOCT00000004606**  26 ---------- ---------- ---------- ---------- ---------- ---------- ---------- ---------- 26

**Spottedgar_ENSLOCT00000004639**  29 ---------- ---------- ---------- ---------- ---------- ---------- ---------- ---------- 29

**Tilapia_ENSONIT00000020351**  27 ---------- ---------- ---------- ---------- ---------- ---------- ---------- ---------- 27

**Hs_TCN1_NP_001053.2**  207 IKADEGSLKN ISIYTKSLVE KILSEKKENG LIGNTFSTGE AMQALFVSSD YYNENDWNCQ QTLNTVLTEI SQGAFSNPNA 286

**Hs_GIF_NP_005133.2**  189 VGSEEGYRSL FGQVLKDIVE KISMKIKDNG IIGDIYSTGL AMQALSVTPE -PSKKEWNCK KTTDMILNEI KQGKFHNPMS 267

**Hs_TCN2_NP_000346.2**  209 -NFNPGRRQR ITMAIRTVRE EILKAQTPEG HFGNVYSTPL ALQFLMTSPM RGAELGTACL KARVALLASL QDGAFQNALM 288

**Medaka_ENSORLT00000019254**  23 ---------- ---------- ---------- ---------- ---------- ---------- ---------- ---------- 23

**Medaka_ENSORLT00000001408**  51 ---------- ---------- ---------- ---------- ---------- ---------- ---------- ---------- 51

**Tilapia_ENSONIT00000013242**  1 ---------- ---------- ---------- ---------- ---------- ---------- ---------- ---------- 1

**Tetradon_ENSTNIT00000017613**  27 ---------- ---------- ---------- ---------- ---------- ---------- ---------- ---------- 27

**Cavefish_ENSAMXT00000002854**  11 ---------- ---------- ---------- ---------- ---------- ---------- ---------- ---------- 11

**Stickleback_ENSGACT00000027033** 27 ---------- ---------- ---------- ---------- ---------- ---------- ---------- ---------- 27

**Platyfish_ENSXMAT00000005953**  49 ---------- ---------- ---------- ---------- ---------- ---------- ---------- ---------- 49

**Amazonmolly_ENSPFOT00000010148** 48 ---------- ---------- ---------- ---------- ---------- ---------- ---------- ---------- 48

**Tilapia_ENSONIT00000006763**  27 ---------- ---------- ---------- ---------- ---------- ---------- ---------- ---------- 27

**Tetraodon_ENSTNIT00000014896**  26 ---------- ---------- ---------- ---------- ---------- ---------- ---------- ---------- 26

**Codfish_ENSGMOT00000005936**  1 ---------- ---------- ---------- ---------- ---------- ---------- ---------- ---------- 1

**Cavefish_ENSAMXT00000003614**  30 ---------- ---------- ---------- ---------- ---------- ---------- ---------- ---------- 30

**Zebrafish_ENSDART00000098273**  30 ---------- ---------- ---------- ---------- ---------- ---------- ---------- ---------- 30

**Zebrafish_ENSDART00000147769**  30 ---------- ---------- ---------- ---------- ---------- ---------- ---------- ---------- 30

**Spottedgar_ENSLOCT00000004606**  26 ---------- ---------- ---------- ---------- ---------- ---------- ---------- ---------- 26

**Spottedgar_ENSLOCT00000004639**  29 ---------- ---------- ---------- ---------- ---------- ---------- ---------- ---------- 29

**Tilapia_ENSONIT00000020351**  27 ---------- ---------- ---------- ---------- ---------- ---------- ---------- ---------- 27

**Hs_TCN1_NP_001053.2**  287 AAQVLPALMG KTFLDINKDS SCVSASGNFN ISADEPITVT PPDSQSYISV NYSVRINE-- ------TYFT NVTVLNGSVF 358

**Hs_GIF_NP_005133.2**  268 IAQILPSLKG KTYLDVPQVT CSPDHEVQPT LPSNPGPG-- -PTSASNITV IYTIN-NQLR GVELLFNETI NVSVKSGSVL 343

**Hs_TCN2_NP_000346.2**  289 ISQLLPVLNH KTYIDLIFPD CLAPRVML-- ----EPAAET IPQTQEIISV TLQV----LS LLP---PYRQ SISVLAGSTV 355

**Medaka_ENSORLT00000019254**  23 ---------- ---------- ---------- ---------- -----DPAPI QIVVK-NSFL EEE---PLAF NSHVAHRGIL 54

**Medaka_ENSORLT00000001408**  51 ---------- ---------- ---------- ---------- -----NLDPI TIVVK-NKFQ GV----KKTY NASVAYRGIL 81

**Tilapia_ENSONIT00000013242**  1 ---------- ---------- ---------- ---------- ------MIPI AIMVK-NTLQ NKP---LQTY KTEVISGGIL 30

**Tetradon_ENSTNIT00000017613**  27 ---------- ---------- ---------- ---------- ------SVPI AVVVQ-NLLH NKP---SLTF TTSTADGGIL 57

**Cavefish_ENSAMXT00000002854**  11 ---------- ---------- ---------- ---------- -----SPYKI SLVVY-NSLT TAK---NLTF STDIAYRGIL 42

**Stickleback_ENSGACT00000027033** 27 ---------- ---------- ---------- ---------- -----GARPI RLSVE-NDLS NIT---PESY FSSVVEGGVL 58

**Platyfish_ENSXMAT00000005953**  49 ---------- ---------- ---------- ---------- -----ESLPI RLTVE-NDLH NMA---PESF SSTVVKEGVL 80

**Amazonmolly_ENSPFOT00000010148** 48 ---------- ---------- ---------- ---------- -----TSLPI RLTVE-NDLS NTA---PESF SSSVVEGGVL 79

**Tilapia_ENSONIT00000006763**  27 ---------- ---------- ---------- ---------- -----GSLSI KLSVE-NELS NEP---LKSY SSSVVEGGVL 58

**Tetraodon_ENSTNIT00000014896**  26 ---------- ---------- ---------- ---------- -----AALPL RLSVV-NTLS DMV---PGSY SSSVVEGGVL 57

**Codfish_ENSGMOT00000005936**  1 ---------- ---------- ---------- ---------- --------PI RVSVEGRGLS SEA---TGSY SGSVVEGGVL 29

**Cavefish_ENSAMXT00000003614**  30 ---------- ---------- ---------- ---------- -----KPVPI RVTVK-DEFS AS----SSFF QTSVLEGGVL 60

**Zebrafish_ENSDART00000098273**  30 ---------- ---------- ---------- ---------- ------EIPV KVTIV-NDFT NE----QLSY STTVIQEGLM 59

**Zebrafish_ENSDART00000147769**  30 ---------- ---------- ---------- ---------- -GGVPGQVSI NVVVT-NKFA NE----LNTY PVTAPKGMPI 64

**Spottedgar_ENSLOCT00000004606**  26 ---------- ---------- ---------- ---------- -----GWSPI LLSVR-NAID QKA---PLSF RGSVPYRGSL 57

**Spottedgar_ENSLOCT00000004639**  29 ---------- ---------- ---------- ---------- ----SKWSPI QLSVE-NAIE STP---PLIF KGSVPYRGVL 61

**Tilapia_ENSONIT00000020351**  27 ---------- ---------- ---------- ---------- ------KVQV NVSPK-N--- ------IKTY STSTAYRGSL 51

**Hs_TCN1_NP_001053.2**  359 LSVMEKAQKM NDTIFGFTME -ERSW-GPYI TCIQGLCANN NDRTYWELLS GG-----EPL SQGAGSYVVR NGENLEVRWS 431

**Hs_GIF_NP_005133.2**  344 LVVLEEAQRK NPM-FKFETT M-TSW-GLVV SSINNIAENV NHKTYWQFLS GV-----TPL NEGVADYIPF NHEHITANFT 415

**Hs_TCN2_NP_000346.2**  356 EDVLKKAHEL G----GFTYE TQASLSGPYL TSVMGKAAG- -EREFWQLLR DPN----TPL LQGIADYRPK DGETIELRLV 425

**Medaka_ENSORLT00000019254**  55 LGAMRTLMDS DTN-FKFTYR EDPNY-GPHL ESINGLAGKD ADQTYWELLV MKPDGAITRP DVGIGCYIPS ANEKIIFNFT 132

**Medaka_ENSORLT00000001408**  82 IGAMKRLRKS NAN-FKFTYK EDLNY-GPYL ESINGVPGKT EDHTYWELLV IKPNGSVIIP DVGIGCYIPS PNEQILFNFT 159

**Tilapia_ENSONIT00000013242**  31 LGAMTRLRDS DAG-FTFTFS DNVNY-GPYL ESVNGVTGNN EAHTYWELLA NVTNGGFQRT EVGIGCIIPS PYQQIILNFT 108

**Tetradon_ENSTNIT00000017613**  58 LGGLRRLMKS NAG-FTFGYS EHPDY-GPFL ESVNGLAGSD RDRTYWELLV RTADGRLLRP DVGIGCYVPK PKDQIILNFT 135

**Cavefish_ENSAMXT00000002854**  43 LGAMRKIAAK TND-FKFTIR DDLNY-GPFL VSVNGVAGG- -DHTYWELLS KRANGTIIRP EVGVGCFIPD PDDTVILKYT 118

**Stickleback_ENSGACT00000027033** 59 LSALRRLQET QQD-FKFTVT VDPNF-GLFL ESVNGVAGSE SEQTYWEILS ESF-GEYTRL DVGIGCYQPV ADEHIILRFS 135

**Platyfish_ENSXMAT00000005953**  81 FGALTRLQET QPD-FKFTVT VDPNF-GLFL ESVNGVAGDE NQQTYWEILT ENS-GEYTRL DVGIGCYTPK ADEHIVLRFR 157

**Amazonmolly_ENSPFOT00000010148** 80 FGALTRLQET QPD-FKFTVK VDPNF-GLFL ESVNGVAGDE NEQTYWEILT ENS-GEYTRL DVGIGCYTPK ADEHIVLKFR 156

**Tilapia_ENSONIT00000006763**  59 LGALRRLHDA QHD-FKFTVK EDPNF-GLFL ESVNGVAGNK DEKTYWEILS ESS-GEFNRL DVGIGCYMPK ADEHIVLRYT 135

**Tetraodon_ENSTNIT00000014896**  58 MGALRRLQET QHN-FKFTVK WDPDF-GLFL ESVNGVAGNV HEQTYWEILS ESS-EEHRRI DLGLGCYKPK ANEHIILRFT 134

**Codfish_ENSGMOT00000005936**  30 LGALKRLQQT DPS-FRFTLK EDPDH-GLFL ESVNGVAGSG QAQSYWELLS ASAPGDPARL DAGIGCYKPK AGEHIILRLS 107

**Cavefish_ENSAMXT00000003614**  61 YGALTRLQDS SNG-FKFTVK IDPNL-GLYL ESVNGVAGSE AKHTYWQILS EHD-GTVTKL DVGVGCYQPK KDEHIILKYT 137

**Zebrafish_ENSDART00000098273**  60 FGVLNQLMES NAD-FKFSYT IHHTF-GIYL ESVNGLAGSD EDQTYWELLS EKS-GVVTRL EVGIGCYQVQ RDENLILRFT 136

**Zebrafish_ENSDART00000147769**  65 FGVLNQLQDS N-Q-LNFTYS ISKSY-GIFL ESVNGLAGST ENKTYWELLS KRE-RKTTRL NVGIGCYQPE RNENFIMNFT 140

**Spottedgar_ENSLOCT00000004606**  58 LGAMWRIQQA NSN-FSFETR DDINY-GPYL VSVNGVAGND TAHTYWQLLR YPK----TPL DRGVGCYIPK PNEHIILNFT 131

**Spottedgar_ENSLOCT00000004639**  62 LGAMLRIQQE NSN-FRFETR DDINY-GPYL VSVNGVAGND TAHTYWQLLR YPN----MPL DRGVGCYIPG ENEHIILRFT 135

**Tilapia_ENSONIT00000020351**  52 FGGLTRLKYS NQG-FNFQYI PNDDY-GPFL QSVNGLAGN- -SSYYWQLLS GK-----TPL DVGMGCYLPT ANEVVTLKYT 122

**Hs_TCN1_NP_001053.2**  432 KY------ 433

**Hs_GIF_NP_005133.2**  416 QY------ 417

**Hs_TCN2_NP_000346.2**  426 SW------ 427

**Medaka_ENSORLT00000019254**  133 KW------ 134

**Medaka_ENSORLT00000001408**  160 KW------ 161

**Tilapia_ENSONIT00000013242**  109 VW------ 110

**Tetradon_ENSTNIT00000017613**  136 RW------ 137

**Cavefish_ENSAMXT00000002854**  119 TW------ 120

**Stickleback_ENSGACT00000027033** 136 TWRRQ--- 140

**Platyfish_ENSXMAT00000005953**  158 TWNSTTVE 165

**Amazonmolly_ENSPFOT00000010148** 157 TWKSTTEE 164

**Tilapia_ENSONIT00000006763**  136 TWSPQQ-- 141

**Tetraodon_ENSTNIT00000014896**  135 KLQPR--- 139

**Codfish_ENSGMOT00000005936**  108 TWSKD--- 112

**Cavefish_ENSAMXT00000003614**  138 TWTKE--- 142

**Zebrafish_ENSDART00000098273**  137 TWATKK-- 142

**Zebrafish_ENSDART00000147769**  141 TWA----- 143

**Spottedgar_ENSLOCT00000004606**  132 TWDNLKRH 139

**Spottedgar_ENSLOCT00000004639**  136 TWD----- 138

**Tilapia_ENSONIT00000020351**  123 KI------ 124

**Supplementary Fig.1. A -** Sequence alignment of holostean and teleost Tcn-like sequences with the 3 human cobalamin binders.

**
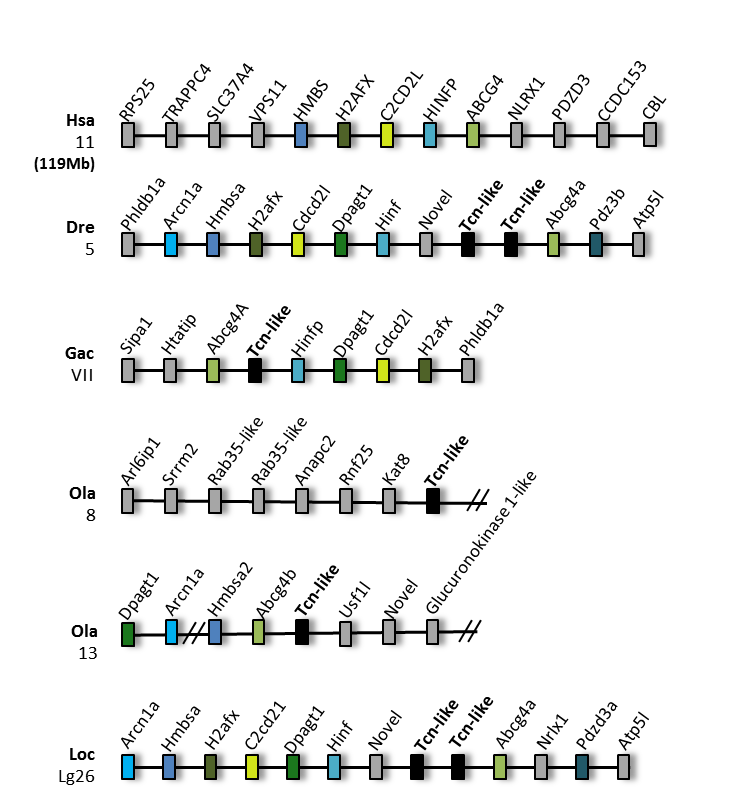
**

**Supplementary Fig. 1. B - S**ynteny maps of the Tcn-like *locus* in fish and the orthologous *locus* in human, Hsa – *H. sapiens*; Dre – *D. rerio*; Gac – *G. aculeatus*; Ola – *O. latipes* and Loc- *L. oculatus.*

**
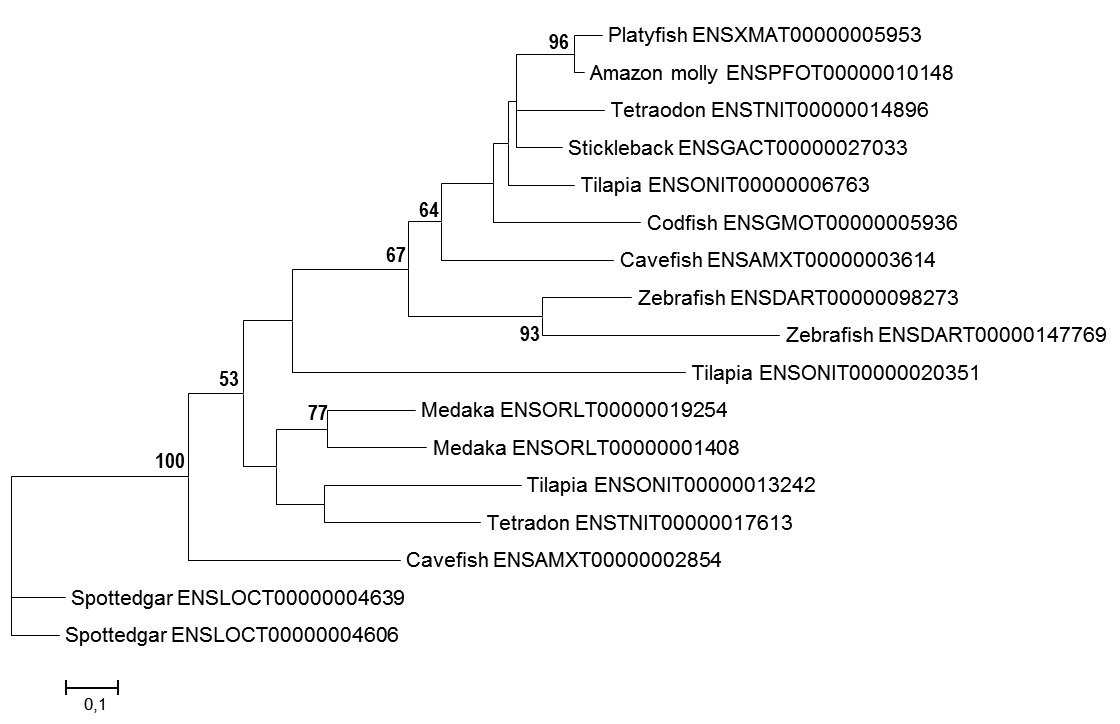
**

**Supplementary Fig. 1. C -** Maximum Likelihood tree of Tcn-like genes, with 100 bootstrap replicates. Bootstrap values below 50 were removed.

**
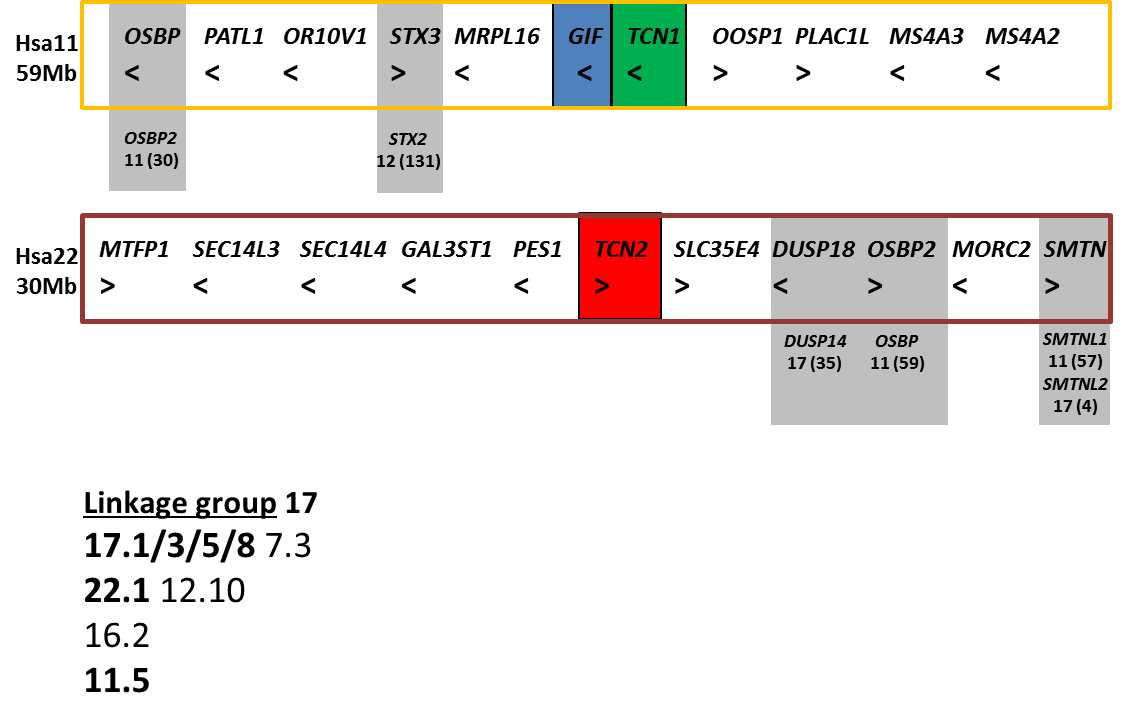
**

**Supplementary Fig. 2.** Paralogy analysis of the *Tcn1*, *Gif* and *Tcn2* human *loci* below (grey) corresponding paralogues of neighboring genes all mapping to the ancestral LG17 (see Putnam et al., 2008 for details of chromosome coordinates of linkage group 17).


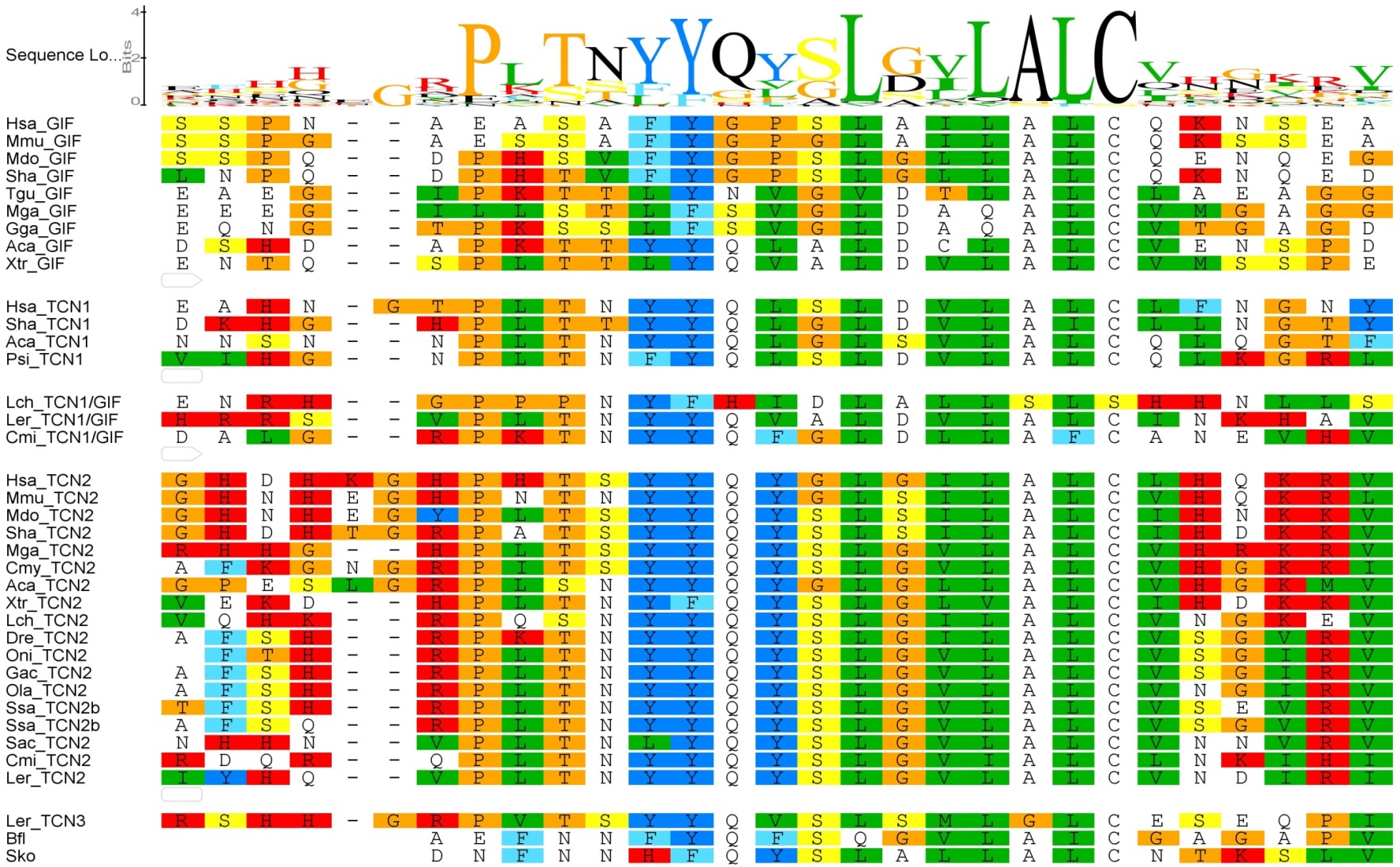

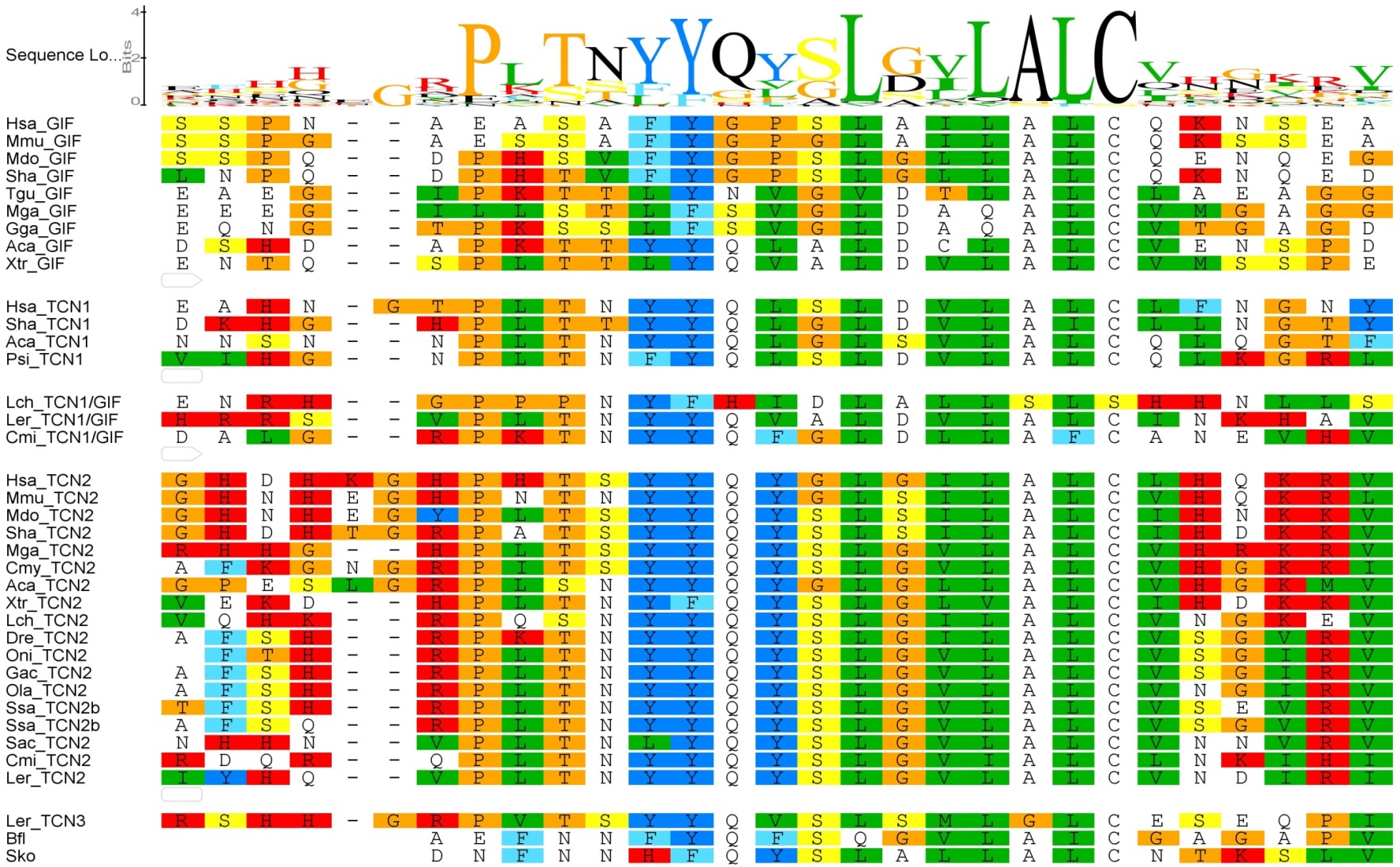

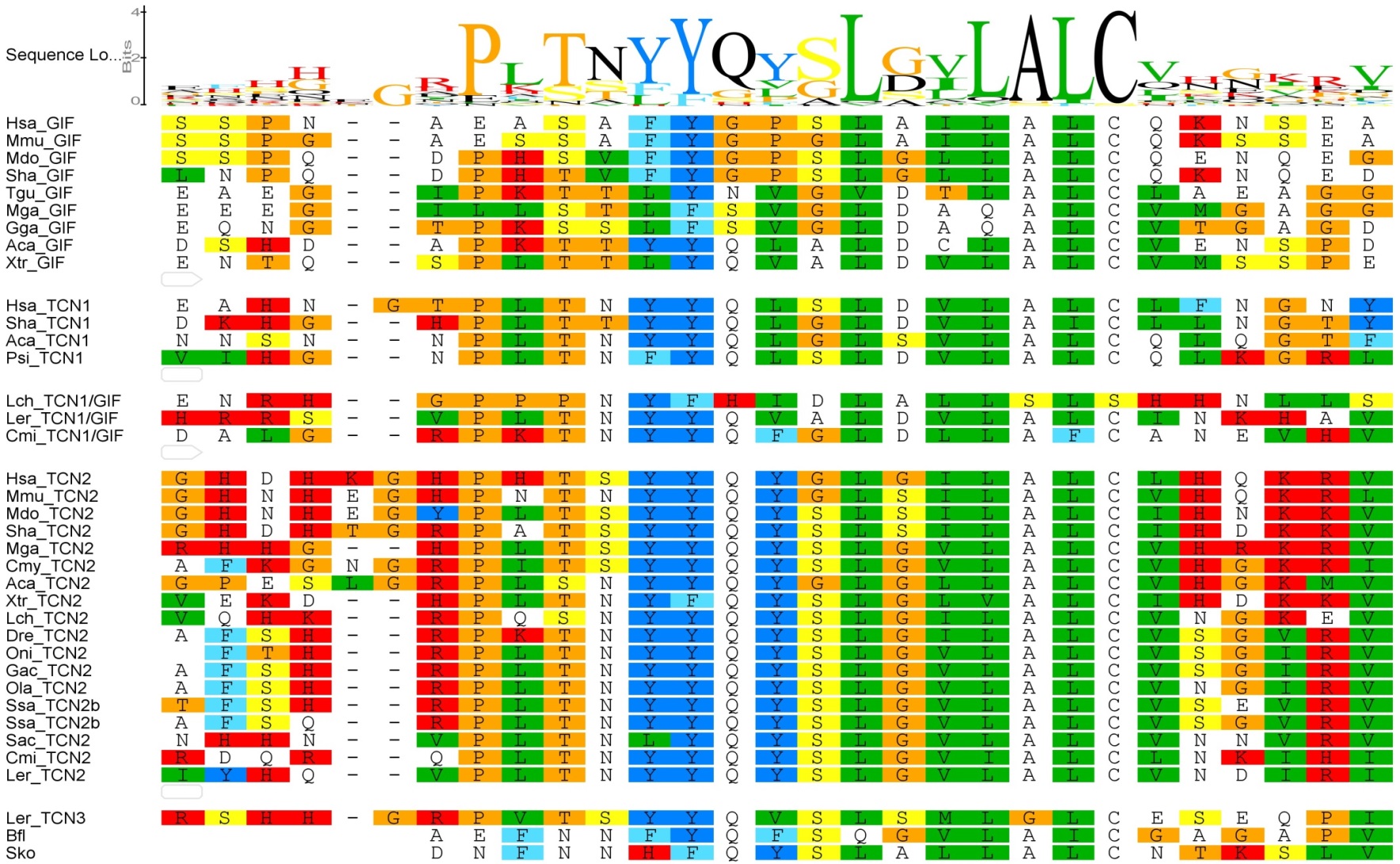

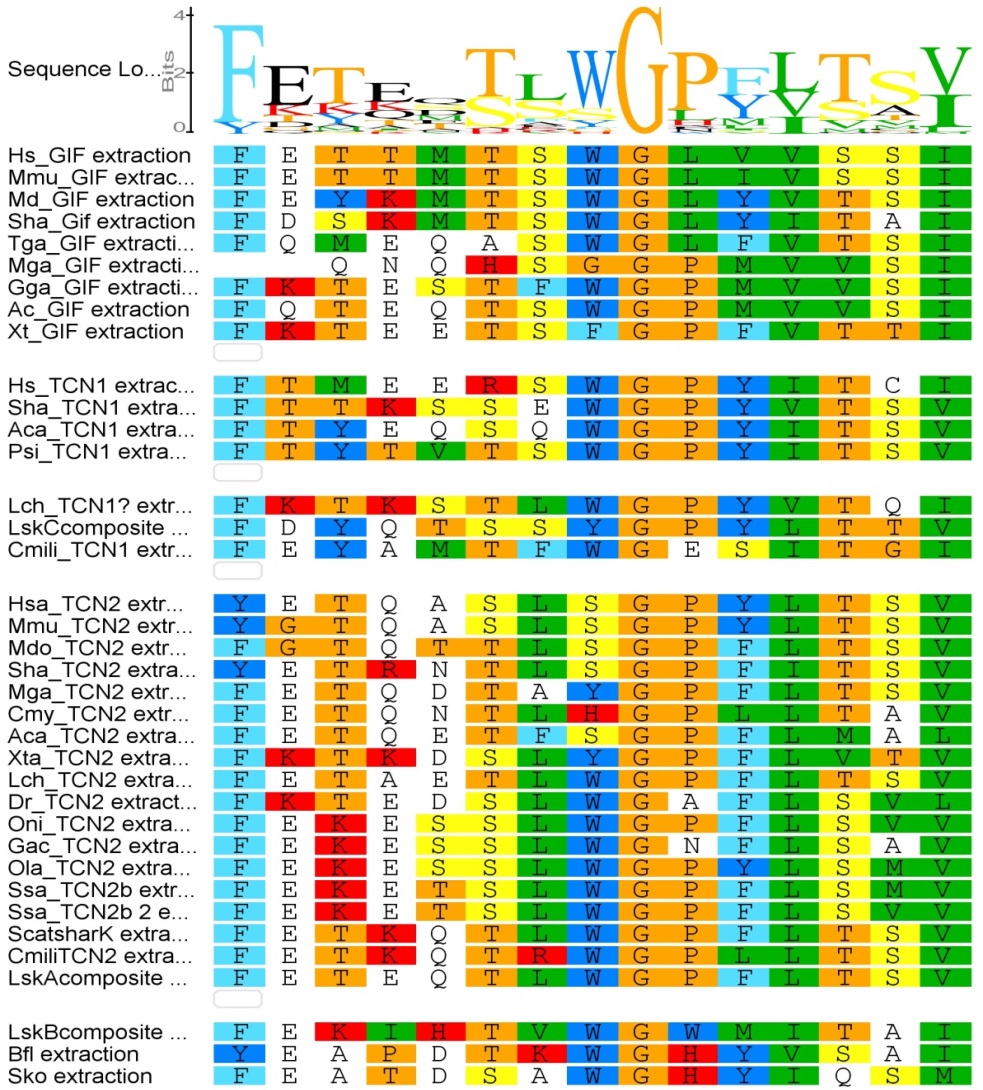


**A**

**B**

**Supplementary Fig. 3.** Motif sequence alignment of *Tcn1*, *Gif* and *Tcn2*. (A) The TNNYQ motif in the α-domain suggested to form 4 hydrogen bonds with the corrinoid moiety in human *Tcn1* contributing for a high affinity towards Cbl;(B) the bulky hydrophobic residue region suggested to compensate for the missing nucleotide in corrinoids contributing for a lower specificity in binding.

**
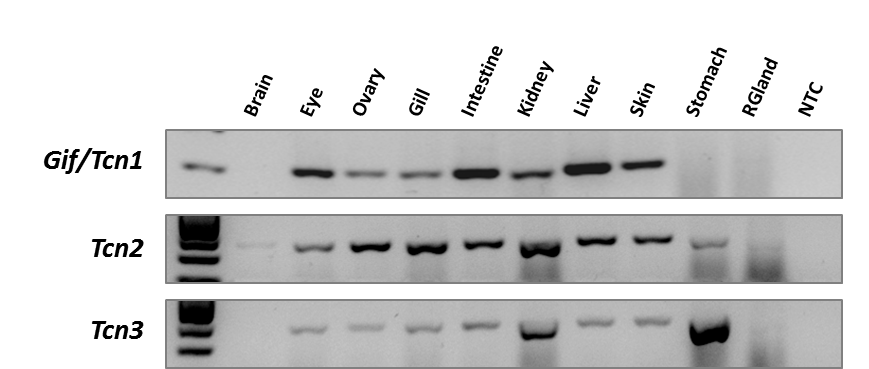
**

**Supplementary Fig. 4.** *Tcn1/Gif, Tcn2* and *Tcn3* gene expression analysis in a tissue panel of *L. erinacea*.


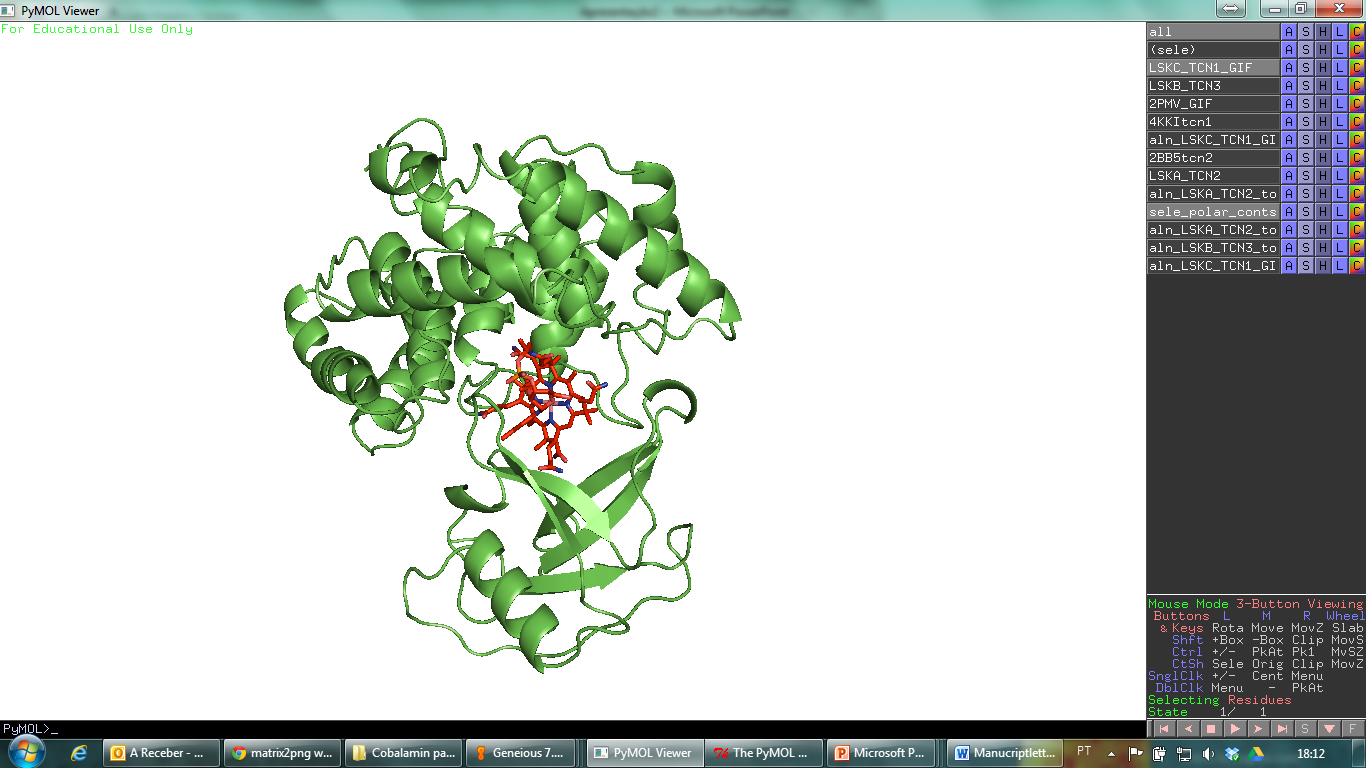

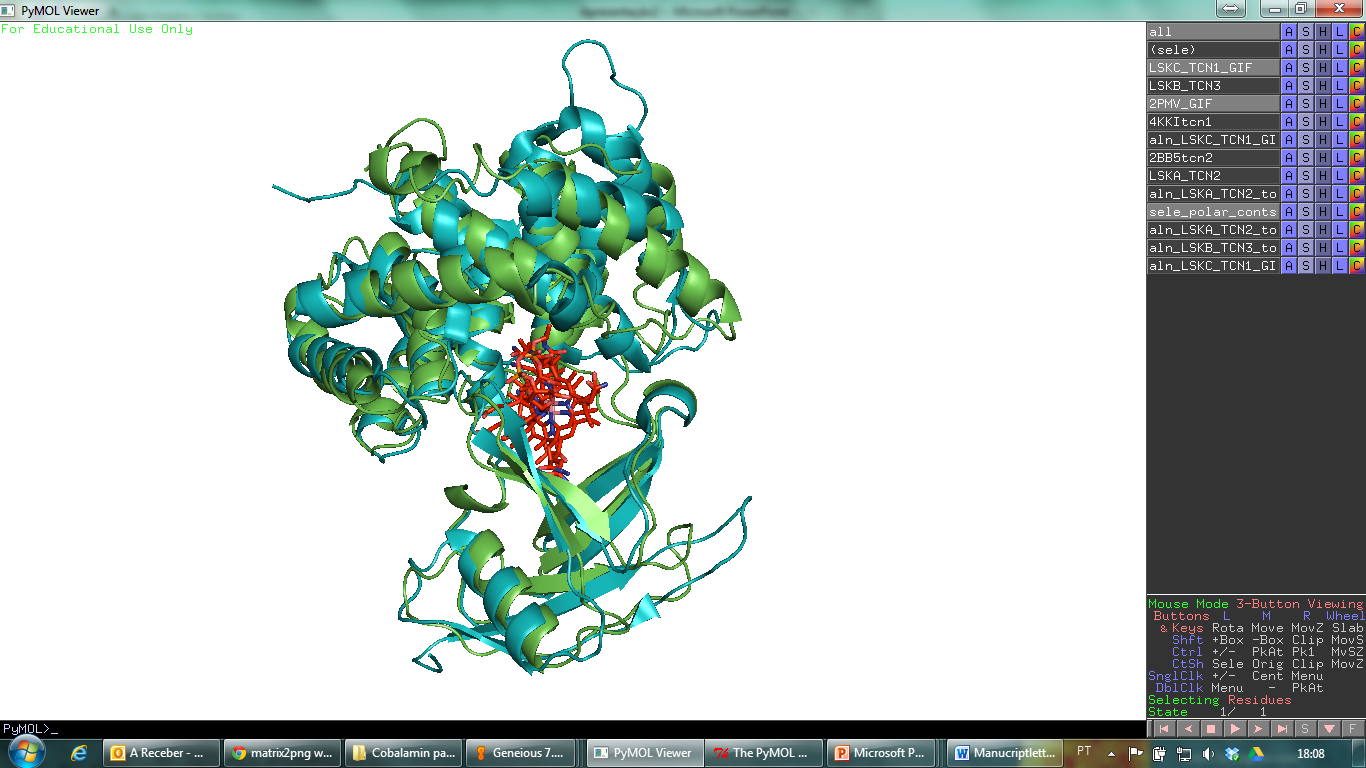


**A**


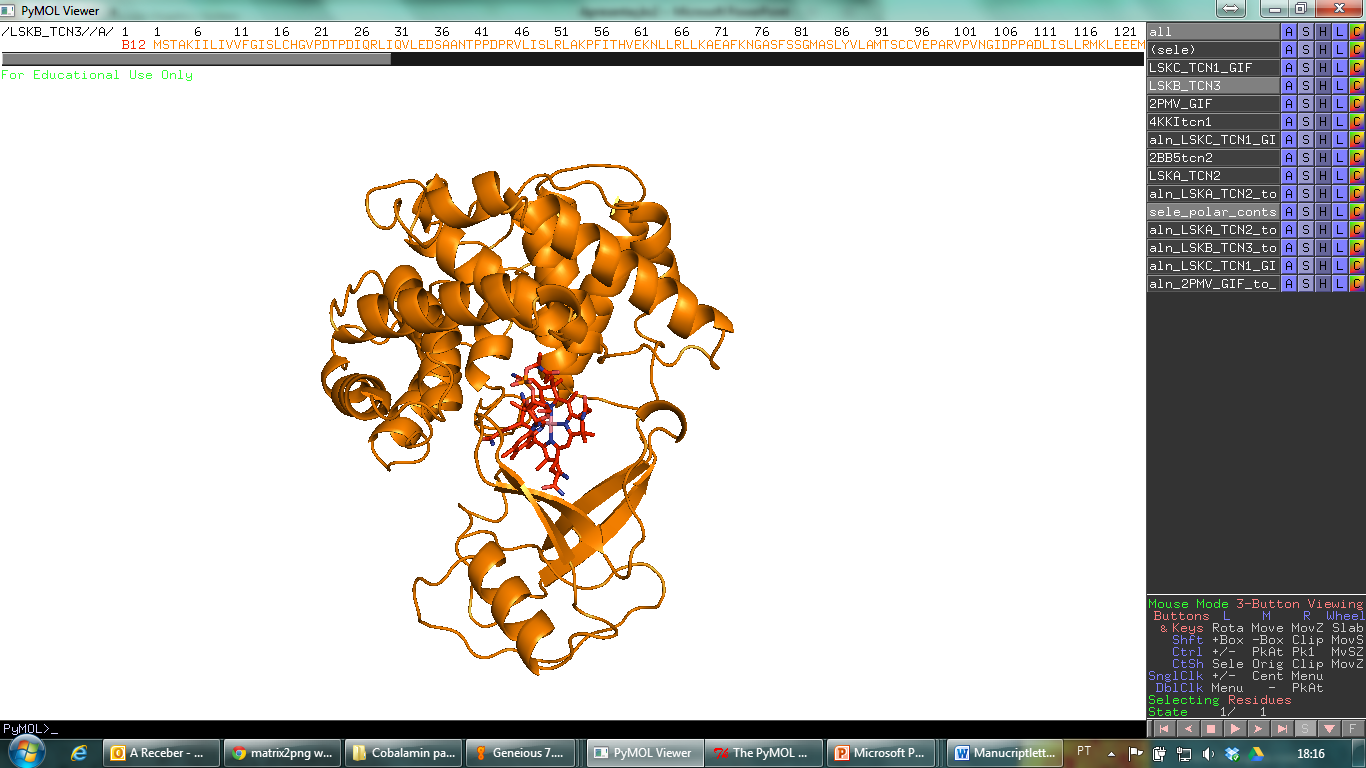

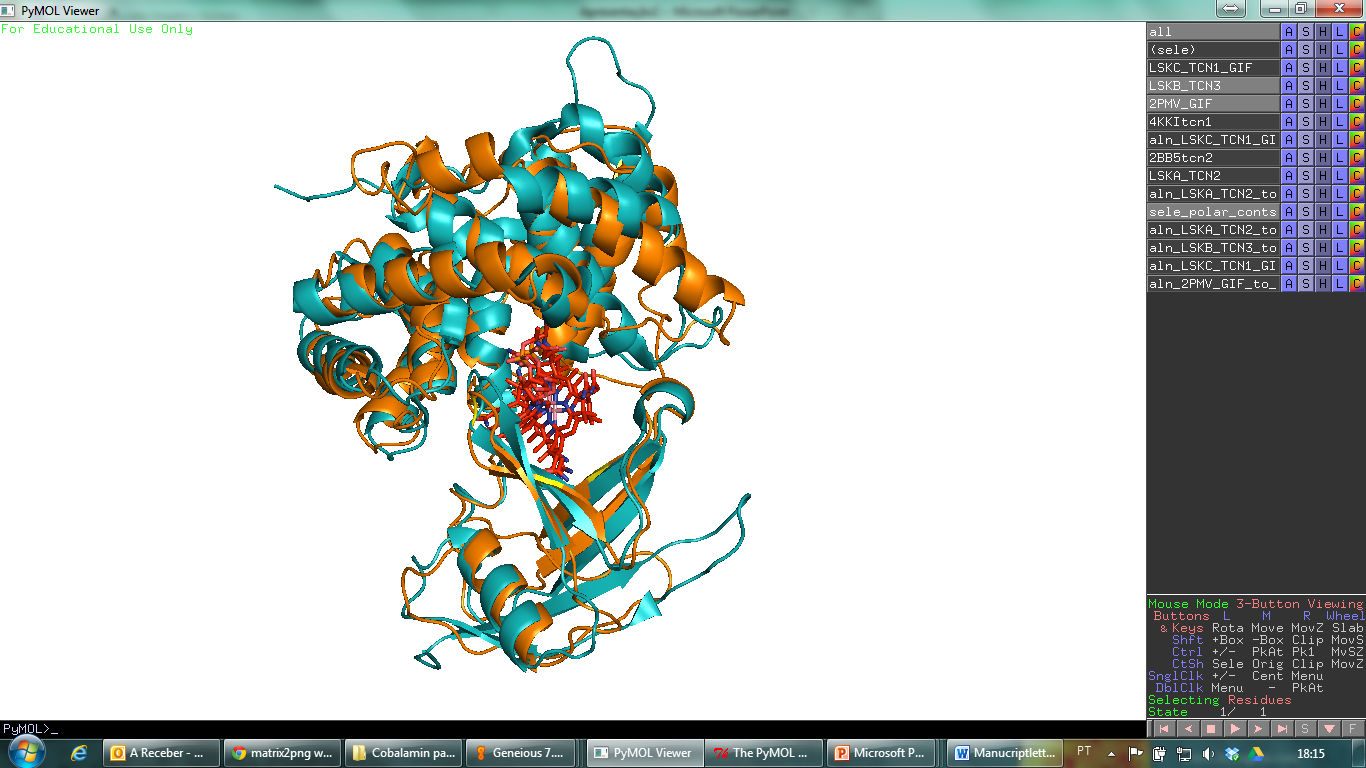


**C**


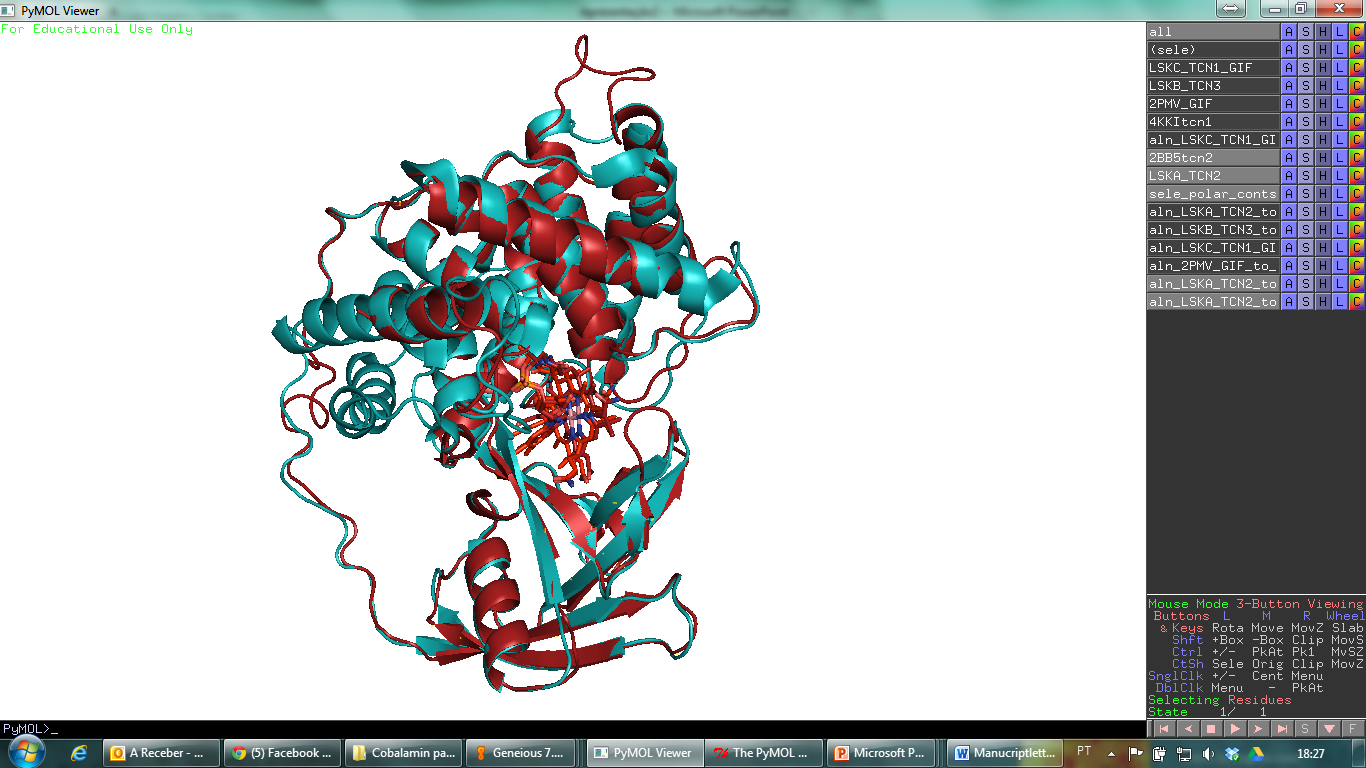

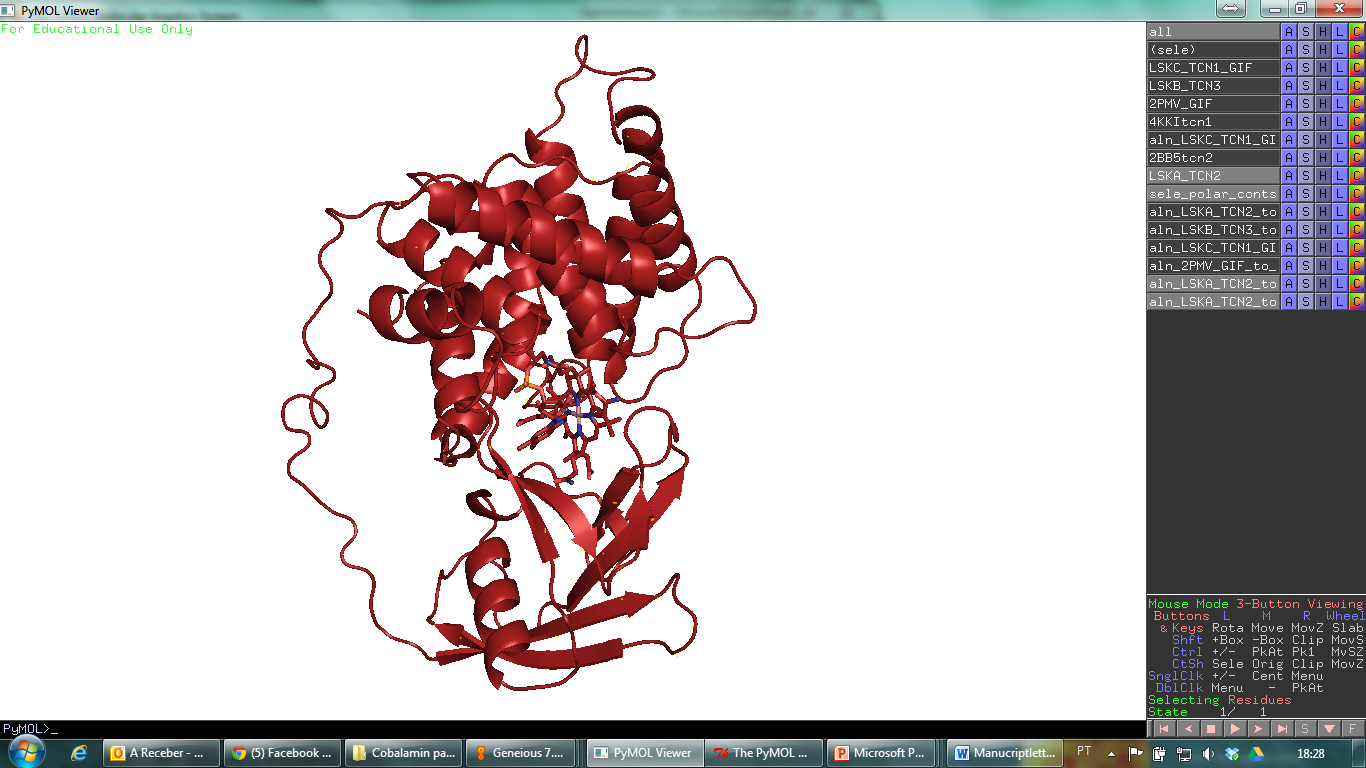


**B**

BS-score = 1.41

BS-score = 1.46

BS-score = 1.38

**Supplementary Fig. 5**. Structural analysis of the *L. erinacea Tcn1/Gif*, *Tcn2* and *Tcn3* 3D models displaying a highly conserved 3D structure. All models contain a B12 molecule (red) in the predicted binding site. Binding Site Score above 1 reflects significant local match between the predicted and template binding site. **(A)** On the right Tcn1/Gif 3D model, on the left structural alignment with 2PMV crystal structure **(B)** Tcn2 3D model on the right, structural alignment with 2BB5 crystal structure on the left and **(C)** Tcn3 3D model on the right and structural alignment with crystal structure 2PMV on the left.
